# Supplementary material for: Identification of QTL for barley grain size
Source: PeerJ. 2021 Apr 29;9:e11287. doi: 10.7717/peerj.11287 (PMC8088763; doi:10.7717/peerj.11287)
Supplement: Table S1 [file peerj-09-11287-s001.docx]

**Table S1. Cloned grain size related genes in rice and the orthologs in Barley**

| **Gene name** | **Gene ID** | **Barley orthologs** | **Ortholog physical location** | **QTL** | **QTL zone** | **References** |
| --- | --- | --- | --- | --- | --- | --- |
| *OsPPKL1* | *Os03g0646900* | - | - | - | - | (Zhang et al., 2012) |
| *OsGs5* | *Os05g09520* | HORVU2HG0042890 | 366,292,283-366,294,494 | QGl.NaTx-1H | 256,409,491-431,648,755 | (Li et al., 2011) |
| *OsSPL16* | *Os08g0531600* | - | - | - | - | (Wang et al., 2012) |
| *Big Grain 1* | *Os03g0175800* | - | - | - | - | (Liu et al., 2015a) |
| *OsBZR1* | *Os07g0580500* | - | - | - | - | (Fang et al., 2020) |
| *Small Grain I* | *Os02g0787300* | HORVU2HG0381450 | 298,580,410-298,583,278 | QGl.NaTx-5H | 25,253,247-334,275,373 | (Duan et al., 2014) |
| *OsMAPK6* | *Os06g0154500* | HORVU2G0040860 | 348,324,310-348,327,386 | QGl.NaTx-1H | 256,409,491-431,648,755 | (Liu et al., 2015b) |
| *TGW6* | *Os06g0623700* | - | - | - | - | (Ishimaru et al., 2013) |
| *GSE5* | *Os05g0187500* | - | - | - | - | (Li et al., 2011) |

|  |  |  |  |  |
| --- | --- | --- | --- | --- |
