# Supplementary material for: Identification of QTL for barley grain size
Source: PeerJ. 2021 Apr 29;9:e11287. doi: 10.7717/peerj.11287 (PMC8088763; doi:10.7717/peerj.11287)
Supplement: Table S2 [file peerj-09-11287-s002.docx]

Table S2 QTLs for grain size traits detected in the DH population of Naso Nijo × TX9425 from different environments

| **Trait** | **Location** | **Linkage**  **group** | **QTL name** | **2_LOD interval** | **Nearest marker** | **Position**  **(cM)** | **LOD** | **R^2^ (%)** | **Additive**  **effect** |
| --- | --- | --- | --- | --- | --- | --- | --- | --- | --- |
| GL | HZ07 | 1H | Gl1.1 |  | 3256408D2 | 13.53 | 13.82 | 19.9 | -0.1 |
|  |  | 2H | Gl1.2 |  | 3267579D2 | 55.56 | 4.98 | 6.2 | -0.055 |
|  |  | 2H | Gl1.3 |  | 8653740S3 | 63.25 | 7.38 | 9.5 | 0.16 |
|  |  | 3H | Gl1.4 |  | GBM1059 | 132.559 | 7.36 | 9.5 | 0.068 |
|  |  | 3H | Gl1.5 |  | 100013392D5 | 47.57 | 3.07 | 3.7 | -0.04 |
|  |  | 5H | Gl1.6 |  | 3927889S1 | 71.41 | 8.84 | 11.7 | -0.075 |
|  | YC07 | 1H | Gl2.1 |  | 3396481S2 | 70.49 | 6.3 | 15.2 | -0.11 |
|  |  | 2H | Gl2.2 |  | 3663277S2 | 10.75 | 5.42 | 12.8 | -0.1 |
|  |  | 5H | Gl2.3 |  | 3397656D5 | 29.1 | 2.67 | 6.1 | -0.07 |
|  | BS07 | 1H | Gl3.1 |  | 5253356D1 | 103.82 | 6.8 | 17.3 | -0.12 |
|  |  | 2H | Gl3.2 |  | 9772745D2 | 10.87 | 5.31 | 13.2 | 0.11 |
|  | HZ08 | 1H | Gl4.1 |  | 8651385D1 | 72.38 | 7.58 | 15.1 | -0.1 |
|  |  | 2H | Gl4.2 |  | 3266106D2 | 9.21 | 10.95 | 23.1 | -0.12 |
|  |  | 3H | Gl4.3 |  | 6283018S3 | 58.43 | 6.48 | 12.7 | 0.24 |
|  | YC08 | 1H | Gl5.1 |  | 8651385D1 | 72.38 | 6.36 | 11.8 | -0.09 |
|  |  | 2H | Gl5.2 |  | 5257304S2 | 6.3 | 14.49 | 30.7 | -0.15 |
|  |  | 3H | Gl5.3 |  | 6283018S3 | 58.43 | 5.38 | 9.8 | 0.23 |
|  | BS08 | 2H | Gl6.1 |  | 5242635D2 | 54.96 | 4.51 | 12.3 | -0.08 |
|  |  | 2H | Gl 6.2 |  | 4017221S2 | 14.38 | 3.75 | 10.1 | -0.07 |
|  | HZ11 | 1H | Gl7.1 |  | 3397573D1 | 86.69 | 7.34 | 8.2 | -0.069 |
|  |  | 2H | Gl 7.2 |  | 3258917D2 | 8.04 | 12.25 | 26.2 | 0.12 |
|  |  | 3H | Gl 7.3 |  | 100000110D3 | 51.35 | 6.96 | 13.7 | 0.18 |
|  | YC11 | 1H | Gl8.1 |  | 6281540S1 | 70.25 | 7.3 | 11.7 | 0.11 |
|  |  | 2H | Gl8.2 |  | 6258917D2 | 8.04 | 15.23 | 27.9 | 0.15 |
|  |  | 3H | Gl8.3 |  | 6283018S3 | 58.43 | 12.7 | 22.3 | 0.37 |
| GW | HZ07 | 2H | Gw1.1 |  | 3265235D2 | 8.32 | 5.29 | 15.3 | 0.03 |
|  | YC07 | 7H | Gw2.1 |  | 7750755D7 | 14.06 | 3.02 | 9 | -0.02 |
|  | BS07 | 1H | Gw3.1 |  | Bmag0211 | 79.724 | 4.21 | 11.2 | -0.027 |
|  |  | 2H | Gw3.2 |  | 7228088D2 | 14.38 | 4.14 | 11 | 0.027 |
|  | HZ08 | 2H | Gw4.1 |  | 5250344D2 | 4.11 | 3.89 | 10.3 | 0.025 |
|  |  | 3H | Gw4.2 |  | 3274641D3 | 114.65 | 3.41 | 9 | 0.023 |
|  | YC08 | 1H | Gw5.1 |  | 3269492D1 | 66 | 3.61 | 8.6 | 0.02 |
|  |  | 2H | Gw5.2 |  | 3273486D2 | 13.9 | 6.13 | 15.2 | -0.028 |
|  |  | 3H | Gw5.3 |  | 100000029D3 | 51.63 | 3.19 | 7.6 | 0.045 |
|  | BS08 | 2H | Gw6.1 |  | 5250553D2 | 5.42 | 7.45 | 19.9 | -0.12 |
|  |  | 2H | Gw6.2 |  | 3266749D2 | 5.38 | 5.58 | 14.5 | 0.11 |
|  | HZ11 | 2H | Gw7.1 |  | 3270380S2 | 11.26 | 12.83 | 25.6 | -0.042 |
|  |  | 4H | Gw7.2 |  | 3263207D4 | 35.91 | 4.94 | 8.6 | 0.025 |
|  |  | 5H | Gw7.3 |  | 4331017D5 | 46.53 | 5.66 | 10 | 0.026 |
|  |  | 5H | Gw7.4 |  | 3268621D5 | 129.65 | 3.28 | 5.6 | 0.019 |
|  | YC11 | 1H | Gw8.1 |  | 4170979D1 | 65.7 | 5.89 | 11.6 | 0.028 |
|  |  | 2H | Gw8.2 |  | 3263995D2 | 5.38 | 4.61 | 8.9 | -0.024 |
|  |  | 4H | Gw8.3 |  | 3263207D4 | 35.91 | 3.76 | 7.2 | 0.022 |
|  |  | 5H | Gw8.4 |  | 3429564D5 | 83.4 | 9.47 | 19.8 | 0.033 |

Abbreviations for traits/sites are shown in Table 1.

The position is that of the nearest marker; R^2^ means percentage genetic variance explained by the nearest marker.
